# Use of Anticoagulants and Antiplatelet Agents in Stable Outpatients with Coronary Artery Disease and Atrial Fibrillation. International CLARIFY Registry

**DOI:** 10.1371/journal.pone.0125164

**Published:** 2015-04-27

**Authors:** Laurent Fauchier, Nicola Greenlaw, Roberto Ferrari, Ian Ford, Kim M. Fox, Jean-Claude Tardif, Michal Tendera, Ph. Gabriel Steg, CLARIFY Investigators

**Affiliations:** 1 Service de Cardiologie, Centre Hospitalier Universitaire Trousseau and Université François Rabelais, Tours, France; 2 Robertson Centre, University of Glasgow, Glasgow, United Kingdom; 3 Department of Cardiology, University Hospital of Ferrara and Maria Cecilia Hospital, GVM Care&Research, E.S. Health Science Foundation, Cotignola, Italy; 4 NHLI Imperial College, ICMS, Royal Brompton Hospital, London, United Kingdom; 5 Montreal Heart Institute, Université de Montreal, Montreal, Canada; 6 Medical University of Silesia, Katowice, Poland; 7 Université Paris-Diderot, Sorbonne-Paris Cité, Paris, France; 8 INSERM U-1148, Paris, France; 9 Département Hospitalo-Universitaire FIRE, Hôpital Bichat, Assistance Publique—Hôpitaux de Paris, Paris, France; KRH Robert Koch Klinikum Gehrden, GERMANY

## Abstract

**Background:**

Few data are available regarding the use of antithrombotic strategies in coronary artery disease patients with atrial fibrillation (AF) in everyday practice. We sought to describe the prevalence of AF and its antithrombotic management in a contemporary population of patients with stable coronary artery disease.

**Methods and Findings:**

CLARIFY is an international, prospective, longitudinal registry of outpatients with stable coronary artery disease, defined as prior (≥12 months) myocardial infarction, revascularization procedure, coronary stenosis >50%, or chest pain associated with evidence of myocardial ischemia. Overall, 33,428 patients were screened, of whom 32,954 had data available for analysis at baseline; of these 2,229 (6.7%) had a history of AF. Median (interquartile range) CHA_2_DS_2_-VASc score was 4 (3, 5). Oral anticoagulation alone was used in 25.7%, antiplatelet therapy alone in 52.8% (single 41.8%, dual 11.0%), and both in 21.5%. OAC use was independently associated with permanent AF (*p*<0.001), CHA_2_DS_2_-VASc score (*p*=0.006), pacemaker (*p*<0.001), stroke (*p*=0.04), absence of angina (*p=*0.004), decreased left ventricular ejection fraction (*p*<0.001), increased waist circumference (*p*=0.005), and longer history of coronary artery disease (*p*=0.008). History of percutaneous coronary intervention (*p*=0.004) and no/partial reimbursement for cardiovascular medication (*p*=0.01, *p*<0.001, respectively) were associated with reduced oral anticoagulant use.

**Conclusions:**

In this contemporary cohort of patients with stable coronary artery disease and AF, most of whom are theoretical candidates for anticoagulation, oral anticoagulants were used in only 47.2%. Half of the patients received antiplatelet therapy alone and one-fifth received both antiplatelets and oral anticoagulants. Efforts are needed to improve adherence to guidelines in these patients.

**Trial Registration:**

ISRCTN registry of clinical trials: ISRCTN43070564.

## Introduction

Patients with coronary artery disease (CAD) generally receive antiplatelet therapy, while patients with atrial fibrillation (AF) may require oral anticoagulants (OACs) to reduce the risk of thromboembolic events [1,2]. European guidelines on AF have extended indications for anticoagulation for eligible patients at risk of stroke [1]. About 6% of all CAD patients have associated AF, and among AF patients, 20–30% have associated CAD, some of who undergo percutaneous coronary interventions (PCIs) with stent implantation [1–3]. After implantation, the rate of stent thrombosis is high in the absence of antiplatelets [2]; current guidelines therefore recommend the use of an aspirin—P2Y_12_-receptor inhibitor combination therapy for 1–12 months afterwards, and for 1 year in all patients after an acute coronary syndrome (ACS) [1,2]. In subjects with stable CAD and AF, where there is most often the requirement for long-term OAC, there is the need to balance the risk of stroke, coronary events, and recurrent cardiac ischemia against the harm of bleeding caused by combining OAC and antiplatelets [3]. The goal of this analysis from the CLARIFY registry was to describe the prevalence of AF and its antithrombotic management in a contemporary population of patients with stable CAD, overall and in relation to previous myocardial infarction (MI), PCI or coronary artery bypass graft (CABG) surgery.

## Methods

### Ethics Statement

The study is being performed according to the Declaration of Helsinki. Ethics committee approval was obtained in all countries in agreement with local regulations. All patients gave written informed consent, as required by national and local guidelines. The ethical committees are as follows: Comité Independiente de Ética para Ensayos de Farmacología Clínica (Argentina); Comité Independiente de Ética Fundación Rusculleda (Córdoba) (Argentina); Ethik-Kommission der Medizinischen Universität Wien (Austria); Ethik-Kommission der Medizinischen Universität Wien und des Allegemeinen (Austria); Krankenhauses der Stadt Wien Akh (Austria); Bellberry Human Research Ethics Committee (Australia); Comité voor Medische Ethiek of the Universitair Ziekenhuis Antwerpen (Belgium); Comitê de Ética em pesquisa do instituto Nacional de Cardiologia (Brazil); Comissão de Ética para Análise de Projetos de Pesquisa CAPPesq (Brazil); Medical and Health Research and Ethics Committee (Ministry of Health, Brunei Darussalam) (Brunei); National Ethic Committee For Multicenter Trials (Bulgaria); Canadian SHIELD Ethics Review Board (Canada); Ethic Committee of School of Public Health, Fudan University (China); Comité d'évaluation de l'éthique des projets de recherche biomedicale (CEERB) du GHU nord (France); Ethik-Kommission der Bayerischen Landesärztekammer, Mühlbauerstraße 16, 81677 Munich (Germany); Scientific Committee of University General Hopsital of Heraklion (Greece); National Organization for Medicines (Gulf countries); United Arab Emirates; Dubai Health Authority (Gulf countries); Galway (Ireland); Cork (Mercy University Hospital) (Ireland); Cork (Mallow General Hospital) (Ireland); Beaumont Hospital (Ireland); ICGP (Ireland); ICGP (Ireland); HSE North East (Ireland); SJH/AMNCH (Ireland); Tullamore (Ireland); Ethic Committee Of Ferrara Province (Italy); Ethic committee of Shinchon Severance Hospital (Korea); Ethic committee of Seoul Nat'l Univ. Hospital (Korea); Ethic committee of Sejong General Hospita (Korea); Ethic committee of Kyunghee Univ. Hospital (Korea); Ethic committee of Korea Univ. Guro Hospita (Korea); Ethic committee of Samsung Medical Center (Korea); Ethic committee of Asan Medical Center (Korea); Ethic committee of Gangnam Severance Hospital (Korea); Ethic committee of Bundang Seoul Nat'l Hospital (Korea); Ethic committee of NHIC Ilsan Hospital_OH Seungjin (Korea); Ethic committee of Ajou Univ. Hospital (Korea); Ethic committee of Sanggye Baik Hospital (Korea); Ethic committee of Boramae Hospital-ZO Joohee (Korea); Ethic committee of Busan Nat'l Univ. Hospital (Korea); Ethic committee of Inje Univ. Busan Paik Hospital (Korea); Ethic committee of Kosin Univ. Gospel Hospital (Korea); Ethic committee of Daegu Catholic Univ. Hospital (Korea); Ethic committee of Yeungnam Univ. Hospital (Korea); Ethic committee of Kyungsang Univ. Hospital (Korea); Ethic committee of Keimyung Univ. Dongsan Hospital (Korea); Ethic committee of Chonnam Univ. Hospital (Korea); Ethic committee of Wonkwang Univ. Hospital (Korea); Ethic committee of Sooncheonhyang Univ. Chunan Hospital (Korea); Ethic committee of Daejeon Eulji Univ. Hospital_JUNG Kyungtae (Korea); Ethic committee of Presbyterian Hospital (Korea); Ethic committee of Daejeon Eulji Univ. Hospital_LEE Sahng: Premature termination (Korea); Ethic committee of NHIC Ilsan Hospital-JEON Dongwoon (Korea); Ethic committee of Boramae Hospital_KIM Sanghyun (Korea); Ethics Committee off The Research Institute of Cardiology, University of Latvia for Clinical and Physiological Research, and Drug and Pharmaceutical ProductClinical Investigation (Latvia); Lithuanian Bioethics Committee (Lithuania); Medical Research & Ethics Committee (Ministry of Health, Malaysia); Independent Ethics Committee, Sime Darby Medical Centre Subang Jaya (Malaysia); IJN Ethics Committee (IJNEC), National Heart Institute (Malaysia); Medical Ethics Committee, University Malaya Medical Centre (Malaysia); National Committee of Data Protection (Portugal); Ethical committee under the federal department of superintendence in healthcare & social development (Russia); National Guard Health Affairs, King AbdulAziz Medical City, Institutional review board; King Fahd Cardiac Center, King Khalid University Hospital; Prince Sultan Cardiac Center, Riyadh (Saudia Arabia); Parkway Independent Ethics Committee; Etická komisia Bratislavského samosprávneho kraja (Ethic committee of Bratislava Self-Governing Region); Comité Ético de Investigación Clínica del Hospital Clínico San Carlos (Spain); Kantonale Ethikkommision Bern (Switzerland); Siriraj Hospital Faculty of Medicine; Chulalongkorn Hospital Faculty of Medicine; Thammasart Hospital Faculty of Medicine (Thailand); Isle of Wight, Portsmouth & South East Hampshire Research Ethics Committee (UK); Central National ethic committee, Ministry of Health of Ukraine (Kiev, Narodnogo opolchenia (Ukraine); Ministry of Health (Vietman).

CLARIFY is an international, prospective, longitudinal registry of outpatients with stable CAD; 33,428 patients from 45 countries were screened (November 2009 to July 2010) of whom 32,954 had data available for analysis [4]. Eligible patients were adults (≥18 years) with stable CAD, defined as having any of the following criteria: either prior (>3 months) documented MI or revascularization procedure, coronary stenosis >50% on coronary angiography, or chest pain associated with proven myocardial ischemia proven by stress electrocardiogram, stress echocardiography, or myocardial imaging. Patients were excluded if they had been hospitalized within the previous 3 months for cardiovascular disease (including for revascularization), were to undergo planned revascularization, or were unlikely to complete 5-year follow-up. In addition, for patients with documented MI or revascularization procedure, those who had either of these events in the year before inclusion were excluded of the analysis.

Recruitment (of 10–15 outpatients per physician) was performed by cardiologists, internists and primary care physicians, with the aim of consecutive enrolment of eligible patients. Physician selection was based on the best available sources, either local or regional, concerning the epidemiology and medical care data, including available market data and epidemiological surveys. A general target of 25 patients/million inhabitants was used (range 12.5–50) to ensure balanced representation of participating countries.

We focused this analysis on patients diagnosed with AF or atrial flutter associated with stable CAD. Patients with a history of myocardial infarction in the 12 months before inclusion were excluded. AF (paroxysmal, persistent, or permanent), as identified by each investigator, was defined on the electrocardiogram by the replacement of consistent P waves by rapid oscillations or fibrillatory waves that vary in amplitude, shape, and timing, associated with an irregular, often rapid, ventricular response with atrioventricular conduction intact. Individual patient management decisions were decided by each physician.

The CHA_2_DS_2_-VASc score was calculated retrospectively (one point each for a history of heart failure, history of hypertension, age 65–75 years, presence of diabetes mellitus, vascular disease [prior MI, peripheral artery disease, aortic plaque], and sex category [female]; and two points for a prior stroke or TIA or age ≥75 years) [5]. A modified version of the HAS-BLED (hypertension, abnormal renal/liver function, stroke, bleeding history or predisposition, labile international normalized ratio, elderly [>65], drugs/alcohol concomitantly) score, excluding labile international normalized ratio, was calculated to assess bleeding risk, with a score ≥3 indicating high risk [6].

Investigators completed standardized electronic case report forms at baseline. Measures were implemented to ensure data quality: onsite monitoring visits of 100% of the data in 5% of centres selected at random over 5 years of follow-up; regular telephone contact with investigators; and centralized verification of the eCRF for completeness, consistency, and accuracy. Data were collected on patient baseline characteristics, risk factors and lifestyle, medical history, physical condition and vital signs, symptoms, and treatments. We analysed the CLARIFY population by antithrombotic therapy (OAC alone or combined with antiplatelet therapy, and one or more antiplatelet[s] without OAC).

CLARIFY is an observational registry, and the size of the population is not based on treatment comparison; the number of patients in this analysis was dependent on the presence or absence of atrial fibrillation in a cohort of patients with stable CAD.

### Statistical Analysis

All CLARIFY data are collected and analysed at an independent academic statistics centre, the Robertson Centre for Biostatistics, University of Glasgow, UK. Baseline variables are summarized as means (standard deviation) or medians (interquartile range) for continuous data and as counts and percentages for categorical data, and were based on patients in whom data were available. Comparisons between patients with OAC use and those without were made using one-way ANOVA or the Kruskal-Wallis test for continuous variables and Pearson’s Chi-squared test or Fisher’s exact test for categorical variables. A multivariable analysis of independent correlates of OAC use was performed using a logistic regression model. All clinical baseline variables, with the exception of HAS-BLED score, were considered for entry into the model as predictors of OAC use and univariate models for each were produced. The multivariable model was built using a stepwise selection method applied to the remaining significant univariate predictors. A sensitivity analysis on the multivariable model excluding patients from East Asia was also performed to determine whether clinical differences in guidelines for this population affected the multivariable model. All analyses were performed using SAS version 9.2. A significance level of 0.05 was used to test for statistical differences; all tests used were two-sided.

## Results

**Fig 1:**
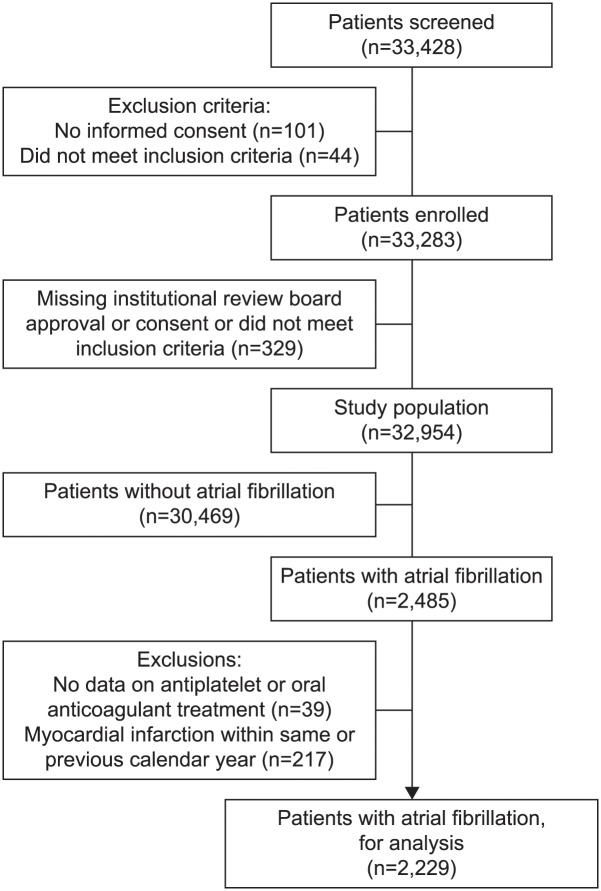
Patient flow chart.

**Fig 2:**
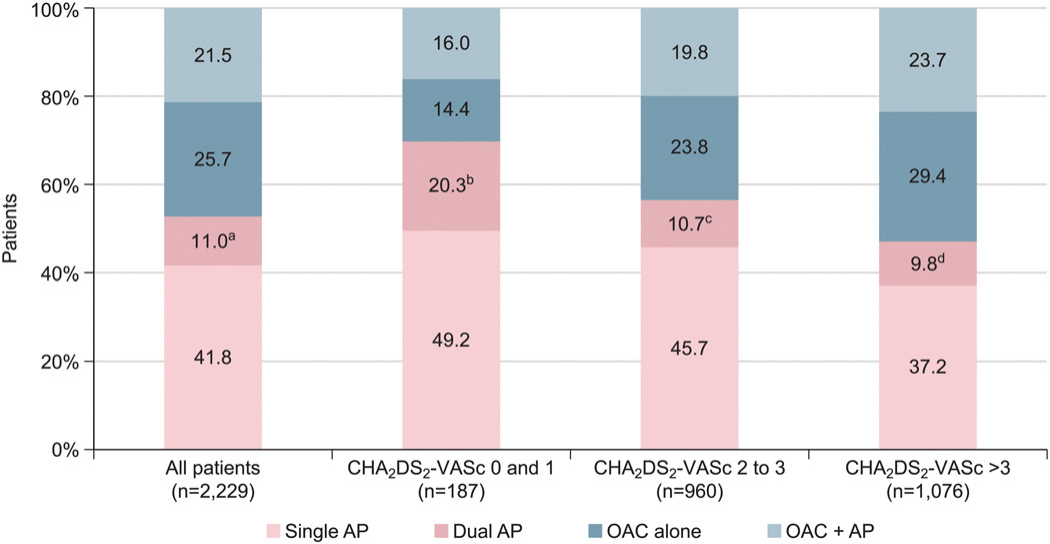
Antithrombotic therapy in patients with coronary artery disease and atrial fibrillation with increasing CHA_2_DS_2_-VASc score. Any antiplatelet therapy alone (single or dual) is therefore: ^a^ 1,178 (52.8%), ^b^ 130 (69.5%), ^c^ 542 (56.4%), ^d^ 505 (46.9%). Abbreviations: AP, antiplatelet; OAC, oral anticoagulant.

Overall, 2,229 of 32,954 patients (6.7%) with stable CAD had a history of known AF, had data on use of antiplatelet or OAC therapy, and had not had a myocardial infarction within the same or previous calendar year (Fig 1). Mean age in these patients was 70 (9) years and median CHA_2_DS_2_-VASc score was 3 (2–5). Known median duration of CAD was 8 (4–13) years and AF was permanent in 41.5% (*n* = 753). OAC alone was used in 25.7% of patients, antiplatelet therapy alone in 52.8% (single 41.8%, dual 11.0%), and both in 21.5%; OAC was thus prescribed to 47.2% of the patients with CAD and AF (Fig 2 and Table 1). Prevalence of AF associated with stable CAD was higher in Europe, North America (Canada), Republic of South Africa, the UK, Russia and Ukraine (ranging from 8.0% to 9.1%) whilst the use of OAC appeared lower in Russia and Ukraine, the Middle East, and East Asia (ranging from 24.4% to 34.2%) (Table 2).

**Table 1:** Use of antithrombotic strategies overall and in CHA_2_DS_2_-VASc subgroups.

Parameter	Subgroup	n	Single AP, n (%)	Dual AP, n (%)	OAC alone, n (%)	OAC+AP, n (%)
**All patients**		2,229	932 (41.8)	246 (11.0) (any antiplatelet therapy alone: 1,178 [52.8])	572 (25.7)	479 (21.5)
**CHA**_**2**_ **DS**_**2**_ **-VASc score**						
	0–1	187	92 (49.2)	38 (20.3) (any antiplatelet therapy alone: 130 [69.5])	27 (14.4)	30 (16.0)
	2–3	960	439 (45.7)	103 (10.7) (any antiplatelet therapy alone: 542 [56.4])	228 (23.8)	190 (19.8)
	>3	1,076	400 (37.2)	105 (9.8) (any antiplatelet therapy alone: 505 [46.9])	316 (29.4)	255 (23.7)

Abbreviations: AP, antiplatelet; OAC, oral anticoagulant therapy.

**Table 2:** Geographic distribution of patients in CLARIFY and use of oral antithrombotic therapy.

Region	Total population (n)	AF subgroup, n (%)	AF patients taking anticoagulation (alone or with antiplatelet), n (%)
**Total**	32,954	2,229	1,051
**Europe**	15,388	1,233 (8.0)	664 (53.9)
**Canada, Republic of South Africa, Australia and the UK**	4,954	451 (9.1)	224 (49.7)
**Russia and Ukraine**	3,026	256 (8.5)	63 (24.6)
**Central and South America**	2,231	80 (3.6)	42 (52.5)
**Middle East**	1,511	38 (2.5)	13 (34.2)
**East Asia**	5,035	160 (3.2)	39 (24.4)
**India**	809	11 (1.4)	6 (54.5)

No adjustment for baseline differences in the populations from different regions.

Abbreviation: AF, atrial fibrillation.

**Fig 3:**
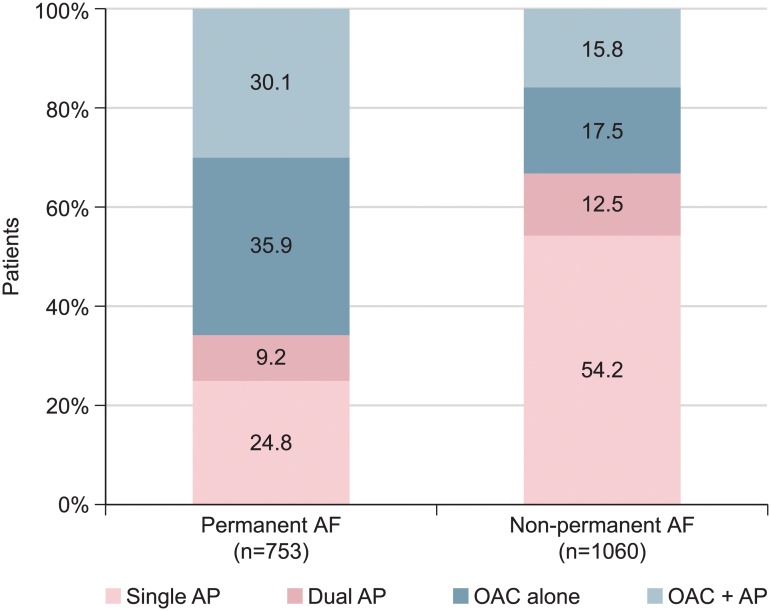
Antithrombotic therapy in patients with coronary artery disease and permanent or non-permanent atrial fibrillation. Abbreviations: AF, atrial fibrillation; AP, antiplatelet; OAC, oral anticoagulant.

Patient baseline characteristics by antithrombotic therapy are shown in Table 3. Non-vitamin K antagonist OACs (dabigatran, rivaroxaban, or apixaban) were not commercially available during the recruitment period. OAC-treated patients were older and more likely to have permanent AF (Fig 3) or be treated with a pacemaker. Half of the patients in both groups had a previous MI, but those on OAC had a lower rate of previous PCI and a higher rate of previous CABG. Fewer patients on OAC had anginal symptoms. The risk of stroke (CHA_2_DS_2_-VASc score) or bleeding (HAS-BLED score) was higher in patients receiving OAC (both *p*<0.001). Patients on OAC therapy had a slightly higher heart rate and lower systolic blood pressure and a lower left ventricular ejection fraction. Patients on OAC were more likely to be reimbursed for cardiovascular medication. Aspirin was given to 39.8% of patients on OAC and to 90.3% of those not treated by OAC. Patients on OAC were less likely to be treated with a thienopyridine (7.9% vs 22.0%) (Table 3).

**Table 3:** Baseline characteristics of patients with stable CAD and atrial fibrillation by antithrombotic therapy.

Parameter	Subgroup	OAC alone or with antiplatelet (n = 1051)	At least 1 antiplatelet (n = 1178)	*p* value
**Age, mean (SD), years** ^a^		71.7 (8.2)	68.6 (9.5)	<0.001
**Men, n (%)** ^a^		847 (80.8)	913 (77.5)	0.055
**BMI, median (IQR), kg/m** ^**2**^ ^b^		28.1 (25.3–31.4)	27.6 (25.0–30.5)	0.0039
**Waist circumference, median (IQR), cm** ^c^		100 (92–109)	99 (90–106)	<0.001
**Education level, n (%)**				0.004
	Primary school or less	328 (31.2)	310 (26.3)	
	Secondary school	471 (44.8)	520 (44.1)	
	College or university	252 (24.0)	348 (29.5)	
**Time since 1st CAD, median (IQR), years**		9 (4–14)	7 (3–12)	<0.001
**Medical history, n (%)**				
	Myocardial infarction^d^	525 (50.0)	591 (50.2)	0.94
	PCI	472 (44.9)	587 (49.8)	0.020
	CABG^e^	392 (37.3)	390 (33.1)	0.038
	Internal cardiac defibrillator	55 (5.2)	28 (2.4)	<0.001
	Pacemaker	154 (14.7)	106 (9.0)	<0.001
	Hospitalization for heart failure	187 (17.8)	127 (10.8)	<0.001
	Stroke	129 (12.3)	68 (5.8)	<0.001
	Permanent AF^f^	497 (58.5)	256 (26.6)	<0.001
	Asthma/COPD	126 (12.0)	149 (12.6)	0.64
	Treated hypertension	824 (78.4)	921 (78.2)	0.90
	Diabetes^d^	309 (29.4)	304 (25.8)	0.056
	Dyslipidaemia	775 (73.7)	925 (78.5)	0.0081
	Peripheral artery disease	191 (18.2)	157 (13.3)	0.0017
**Angina and CCS class, n (%)**				<0.001
	No angina	877 (83.4)	850 (72.2)	
	Class I	50 (4.8)	65 (5.5)	
	Class II	97 (9.2)	191 (16.2)	
	Class III	24 (2.3)	70 (5.9)	
	Class IV	3 (0.3)	2 (0.2)	
**Creatinine concentration, median (IQR), mmol/L** ^g^		0.095 (0.08–0.12)	0.093 (0.08–0.11)	0.045
**Haemoglobin, median (IQR), mmol/L** ^h^		8.6 (8.0–9.3)	8.7 (8.1–9.3)	0.31
**Heart rate (electrocardiogram), mean (SD), beats/min** ^i^		70.9 (14.2)	68.0 (13.0)	<0.001
**Heart rate (palpation), mean (SD), beats/min** ^j^		70.1 (12.7)	68.2 (11.4)	<0.001
**SBP, mean (SD), mmHg** ^a^		130.0 (16.2)	132.2 (16.2)	0.0011
**DBP, mean (SD), mmHg** ^a^		76.3 (9.7)	77.1 (10.4)	0.051
**Left ventricular ejection fraction, mean (SD), (%)** ^k^		52.2 (12.6)	56.2 (11.5)	<0.001
**Vessel disease, n (%)** ^l^				0.050
	0	48 (5.4)	40 (4.1)	
	1	266 (30.2)	343 (35.0)	
	≥2	567 (64.4)	597 (60.9)	
**Baseline medications, n (%)**				
	Aspirin	418 (39.8)	1064 (90.3)	<0.001
	Thienopyridine^m^	83 (7.9)	259 (22.0)	<0.001
	Other antiplatelet^d^	43 (4.1)	110 (9.3)	<0.001
	Beta-blocker	797 (75.8)	895 (76.0)	0.94
	Ivabradine	32 (3.0)	96 (8.1)	<0.001
	Calcium antagonist	282 (26.8)	365 (31.0)	0.03
	Verapamil or diltiazem	76 (7.2)	68 (5.8)	0.16
	ACE inhibitors	575 (54.7)	614 (52.1)	0.22
	Angiotensin II receptor blocker	321 (30.5)	326 (27.7)	0.14
	Lipid-lowering drug	914 (87.0)	1065 (90.4)	0.01
	Long-acting nitrate	221 (21.0)	283 (24.0)	0.09
	Other antianginal agent^e^	111 (10.6)	198 (16.8)	<0.001
	Trimetazidine^e^	68 (6.5)	152 (12.9)	<0.001
	Diuretic^d^	607 (57.8)	506 (43.0)	<0.001
	Other antihypertensive drug^d^	110 (10.5)	104 (8.8)	0.19
	Digoxin and derivative	247 (23.5)	97 (8.2)	<0.001
	Amiodarone/dronedarone^d^	175 (16.7)	223 (18.9)	0.16
	Other antiarrhythmic	38 (3.6)	70 (5.9)	0.01
	Non-steroidal anti-inflammatory drug^e^	44 (4.2)	61 (5.2)	0.27
	Anti-diabetes drug	262 (24.9)	254 (21.6)	0.06
**CHA**_**2**_ **DS**_**2**_ **-VASc score, median (IQR)** ^b^		4 (3–5)	3 (2–4)	<0.001
**CHA**_**2**_ **DS**_**2**_ **-VASc score, n (%)** ^b^				
	0/1/2	214 (20.5)	355 (30.2)	<0.001
	3	261 (25.0)	317 (26.9)	
	4	233 (22.3)	257 (21.8)	
	5	184 (17.6)	159 (13.5)	
	≥6	154 (14.7)	89 (7.6)	
**HAS-BLED score, median (IQR)** ^n^		1 (1–2)	1 (1–1)	<0.001
**HAS-BLED score, n (%)** ^n^				
	<3	958 (91.6)	1153 (98.0)	<0.001
	≥3	88 (8.4)	23 (2.0)	
**ECG rhythm, n (%),** ^f^				<0.001
	Sinus rhythm	270 (31.8)	654 (67.9)	
	AF/flutter	497 (58.5)	256 (26.6)	
	Paced rhythm	83 (9.8)	53 (5.5)	
**LBBB, n (%),** ^o^		85 (10.0)	70 (7.3)	0.035
**Reimbursement of cardiovascular drugs, n (%)** ^b^				<0.001
	Full	525 (50.1)	464 (39.5)	
	Part	367 (35.1)	498 (42.3)	
	Not	155 (14.8)	214 (18.2)	

Data missing for:

^a^3 patients (n = 2,226)

^b^6 patients (n = 2,223)

^c^29 patients (n = 2,200)

^d^1 patient (n = 2,228)

^e^2 patients (n = 2,227)

^f^416 patients (n = 1,813)

^g^499 patients (n = 1,730)

^h^689 patients (n = 1,540)

^i^414 patients (n = 1,815)

^j^4 patients (n = 2,225)

^k^581 patients (n = 1,648)

^l^368 patients (n = 1,861)

^m^5 patients (n = 2224)

^n^7 patients (n = 2,222)

^o^418 patients (n = 1,811).

Abbreviations: BMI, body mass index; CAD, coronary artery disease; CHF, congestive heart failure; COPD, chronic obstructive pulmonary disease; DBP, diastolic blood pressure; ECG, electrocardiogram; IQR, interquartile range; LBBB, left bundle branch block; OAC, oral anticoagulant therapy; SBP, systolic blood pressure; SD, standard deviation.

**Fig 4:**
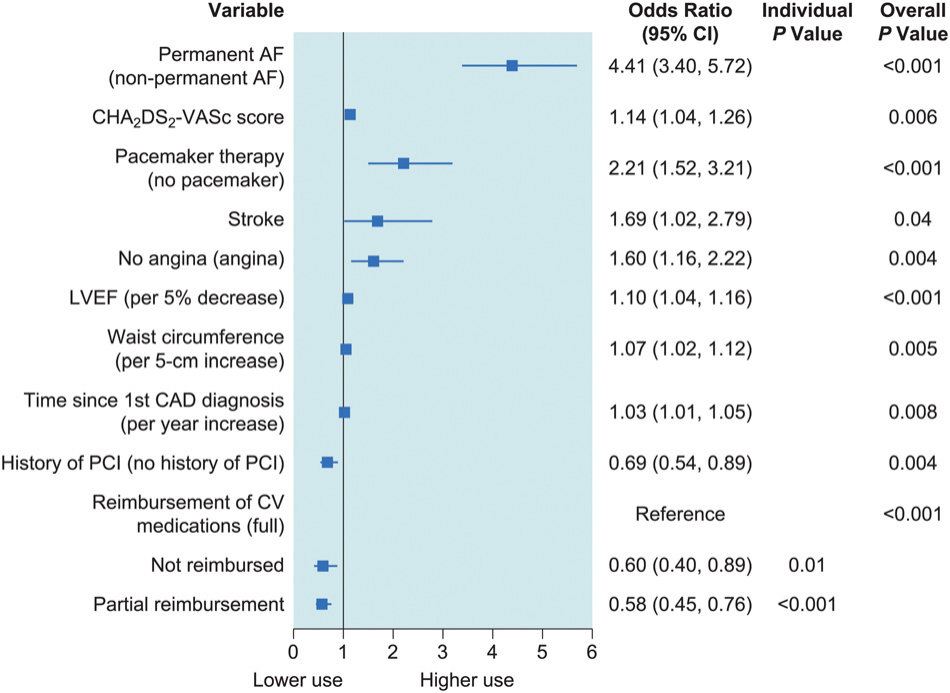
Multivariable logistic regression results for odds of taking any oral anticoagulant therapy (either alone or with an antiplatelet) compared with the antiplatelet alone group. Abbreviations: AF, atrial fibrillation; CAD, coronary artery disease; CI, confidence interval; CV, cardiovascular; OAC, oral anticoagulant; PCI, percutaneous coronary intervention.

In multivariable analysis, OAC use was independently associated with permanent AF, CHA_2_DS_2_-VASc score, pacemaker therapy, stroke, absence of angina, decreased left ventricular ejection fraction, increased waist circumference, and longer history of CAD (Fig 4). Conversely, history of PCI, and no/partial reimbursement for cardiovascular medication were associated with reduced likelihood of OAC use.

## Discussion

In this contemporary international cohort of patients with stable CAD and AF, most of whom are theoretical candidates for anticoagulation, less than 50% of patients received OACs. Half of the patients received antiplatelet therapy alone and one-fifth received antiplatelets plus OAC. This analysis is the first of its kind to evaluate the applicability of the AF guidelines in this subgroup of patients with stable CAD and, to our knowledge, is the largest published dataset in this population, in whom antithrombotic management strategies have been related to baseline clinical characteristics [7].

Limited published evidence is available on the optimal management strategy for patients with CAD and AF [8–13]. Our population reflects a “real-world” scenario for the applicability of current AF guidelines. In our analysis, most of the patients had a CHA_2_DS_2_-VASc score ≥2, with only a minority receiving guideline-recommended antithrombotic therapy. These data suggest under-treatment, but also possible over-treatment, with 21.5% receiving both OAC and antiplatelet therapy, although whether there is benefit or harm in adding antiplatelet therapy to OAC in patients with both AF and coronary artery disease remains debatable [14].

We identified risk factors associated with lack of OAC use. Compared with previous studies on guideline adherence in the more general setting of AF, and based on the 2010 guidelines [1], we found that an even lower percentage of patients with stable CAD and AF was appropriately treated [15,16]. When divided into groups at increasing risk with higher CHA_2_DS_2_-VASc score, patients most at risk of thromboembolism were poorly treated with more OAC. In multivariable analysis, OAC use was independently associated with permanent AF, CHA_2_DS_2_-VASc score, pacemaker therapy, stroke, absence of angina, decreased left ventricular ejection fraction, increased waist circumference, and longer history of CAD (Fig 4). Conversely, history of PCI, and no/partial reimbursement for cardiovascular medication were associated with reduced likelihood of OAC use. Perhaps surprisingly, multivessel disease, history of MI, and coronary artery bypass graft surgery were not independently associated with likelihood of OAC use. Overall, it seems that OAC is less likely to be prescribed in patients with stable CAD and AF when CAD is considered the “primary” condition, as suggested by the lower use among patients with angina or previous PCI, whereas those with heart failure or permanent AF were more likely to receive OAC therapy. OAC use was associated with higher HAS-BLED score, probably because it is often a marker for greater CHA_2_DS_2_-VASc score, the two being correlated. It is also possible that many physicians were unaware of the HAS-BLED score at the time the study was conducted. Antithrombotic treatment differed according to AF type, with paroxysmal and persistent AF being more frequent among patients not receiving OACs. This finding is consistent with previous studies [16,17], and illustrates a gap between everyday clinical practice and the guideline recommendations to provide OAC to patients at risk irrespective of AF type. The speciality of the physician taking care of the patients enrolled (general practitioner versus internist versus cardiologist) may also play a role in the use of OAC and/or antiplatelet therapy in these patients.

Our results emphasize the fact that the individual patient’s thromboembolic risk should first be considered, and their bleeding risk assessed thereafter, but clinicians frequently overestimate bleeding risk [18–20]. All guidelines balance risk of stroke and bleeding. However, a high bleeding risk is not *per se* a contraindication for OAC use and does not challenge our assumption that most if not all patients with stable CAD and AF should be on OAC. Prevention of MI and coronary events is an additional challenge in these patients [13]. Clinicians should probably focus more on the prevention of disabling—and potentially fatal—strokes and severe bleeds, and place less emphasis on minor bleeding events. In the randomized ACTIVE-W trial in patients with AF at risk of stroke, the occurrence of nonfatal strokes was associated with an increased risk of subsequent mortality (hazard ratio 5.58, 95% confidence interval 3.84–8.10, *p*<0.0001), whereas among the major bleeding events, only those also classified as severe increased subsequent mortality (3.35, 2.12–5.27, *p*<0.0001) [21].

The addition of an antiplatelet agent to OAC should be considered in some subgroups with CAD [22]. However, such combination therapy may present a challenge given the increased risk of bleeding associated with these treatments [12]. Combined aspirin with clopidogrel is less effective in preventing stroke than oral anticoagulation alone [23]. Dual antiplatelet therapy has been proposed as an initial option after stent implantation in AF patients at seemingly low risk of thromboembolism in North American guidelines, but OAC alone should be prescribed thereafter [24,25]. Physicians may, however, be reluctant to change antithrombotic management once the patient is stable, which may explain why many patients received long-term antiplatelet therapy in our analysis [26]. Of note, East Asia had guidelines with slightly different thresholds and INR target for OAC use whilst the region contributed substantially to the overall population. Removing East Asia and running a stepwise selection model on this population as a sensitivity analysis provided results that were similar (data not shown).

In a consensus document on the optimal management of antithrombotic therapy in AF patients undergoing PCI/stenting, various antithrombotic strategies have been offered according to the haemorrhagic risk and the clinical setting (elective or acute) [22]. At discharge from hospital, triple antithrombotic therapy (oral anticoagulation, aspirin, and clopidogrel) is recommended, ranging from ≥2 weeks for elective procedures with high haemorrhagic risk up to 6 months for acute coronary syndrome procedures when the bleeding risk is low or intermediate. According to the type of procedure, this regimen is then followed by OAC plus clopidogrel for up to 12 months. Thereafter, warfarin alone is recommended lifelong, and this theoretically should have been the strategy in a majority of our patients treated with PCI [1,22,24].

Current recommendations are based largely on limited evidence from small, single-centre, and retrospectively analysed cohorts [27]. Thus there is a definite need for large-scale registries that reflect patients treated in everyday practice, such as CLARIFY, and prospective clinical studies to determine the optimal management of patients with AF and stable CAD. The development of direct oral anticoagulants, which are more convenient to use and more effective than warfarin in reducing the rates of stroke and systemic embolism with similar rates of major haemorrhage, may be promising for patients with CAD and AF [28,29]. They may improve adherence to guidelines, although further (randomized) studies are needed in this setting.

### Limitations

This study is limited by its observational design. Given the diagnostic methods used with no systematic ambulatory monitoring, the prevalence of paroxysmal AF may have been underestimated, but use of OAC is likely to be even lower in these patients with undiagnosed AF. Although we adjusted for several variables, residual confounding, including variables related to the severity of CAD, may account for some of the observed differences between patients treated with OAC or with antiplatelet therapy. The lack of information on the percentage of patients who had stents implanted and the proportion of bare-metal versus drug-eluting stents represents an issue, as this might have substantially impacted on the prescription as well as duration of antithrombotic therapies, especially of antiplatelets. We did not perform an analysis evaluating whether patients received the same antithrombotic drug during the entire follow-up period or for how long they received it. Nor did we retrieve information on how OAC was monitored or about the quality of anticoagulation control for warfarin. Investigators were free to select the antithrombotic treatments, and such choices are influenced by patient and lesion characteristics: it may be that patients/lesions presumed to be at higher risk of stent thrombosis were preferably treated by antiplatelet therapy to reduce this risk, even in so-called stable CAD after 12 months. Similarly, the expected risk of bleeding may have influenced the type of antithrombotic treatment prescribed, although this does not clearly appear in our multivariable analysis. The lack of use of OAC therefore represents a missed opportunity to prevent an adverse event in patients with AF, but it is possible that operators succeed in equalizing risks and benefits in different patients with CAD and AF by adequate selection of PCI with or without stenting and antithrombotic treatment among different patient groups.

## Conclusion

In this contemporary international cohort of patients with stable CAD and AF, most of whom are theoretical candidates for anticoagulation, OACs were used in a minority, indicating a gap between guidelines for AF and everyday clinical practice.
